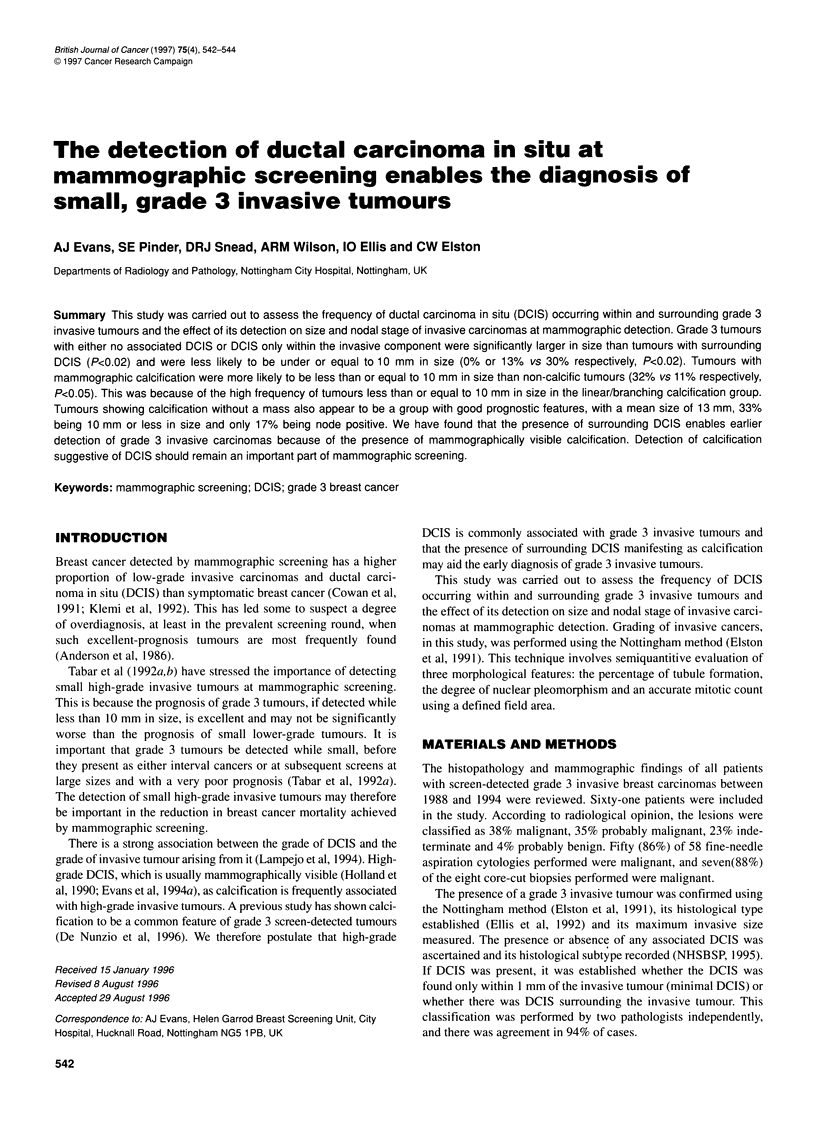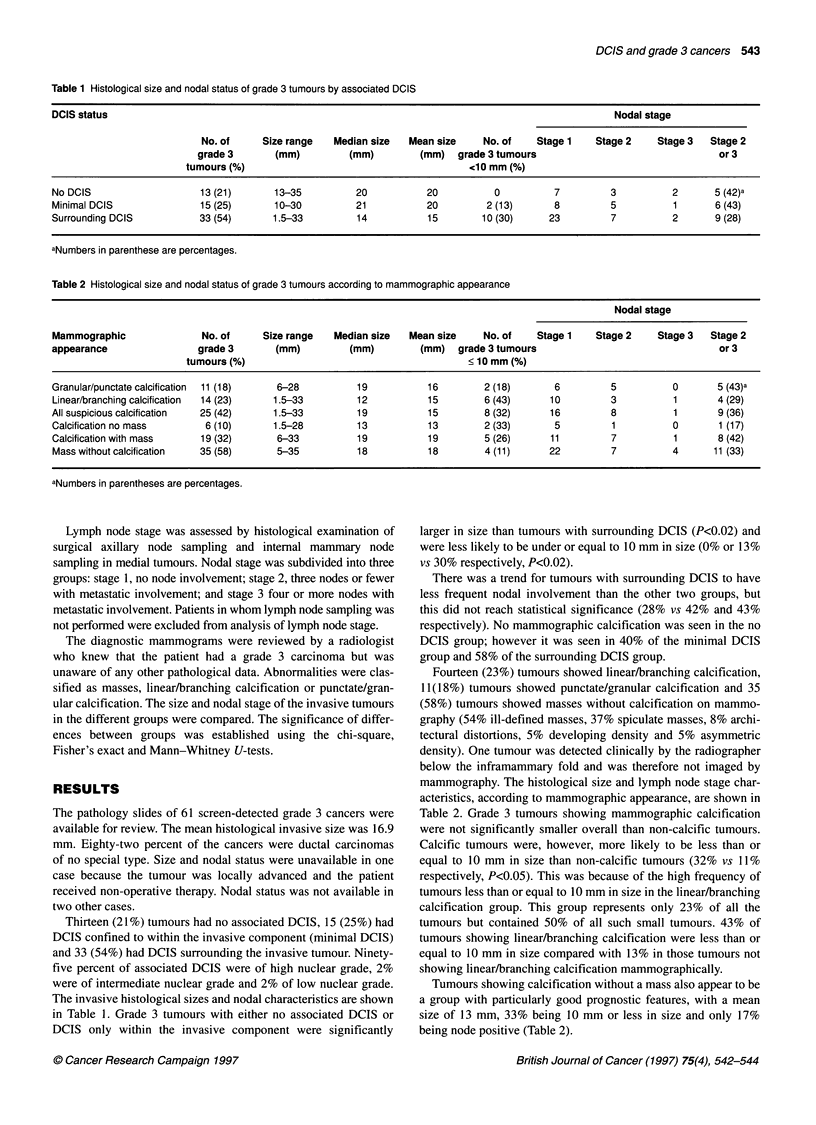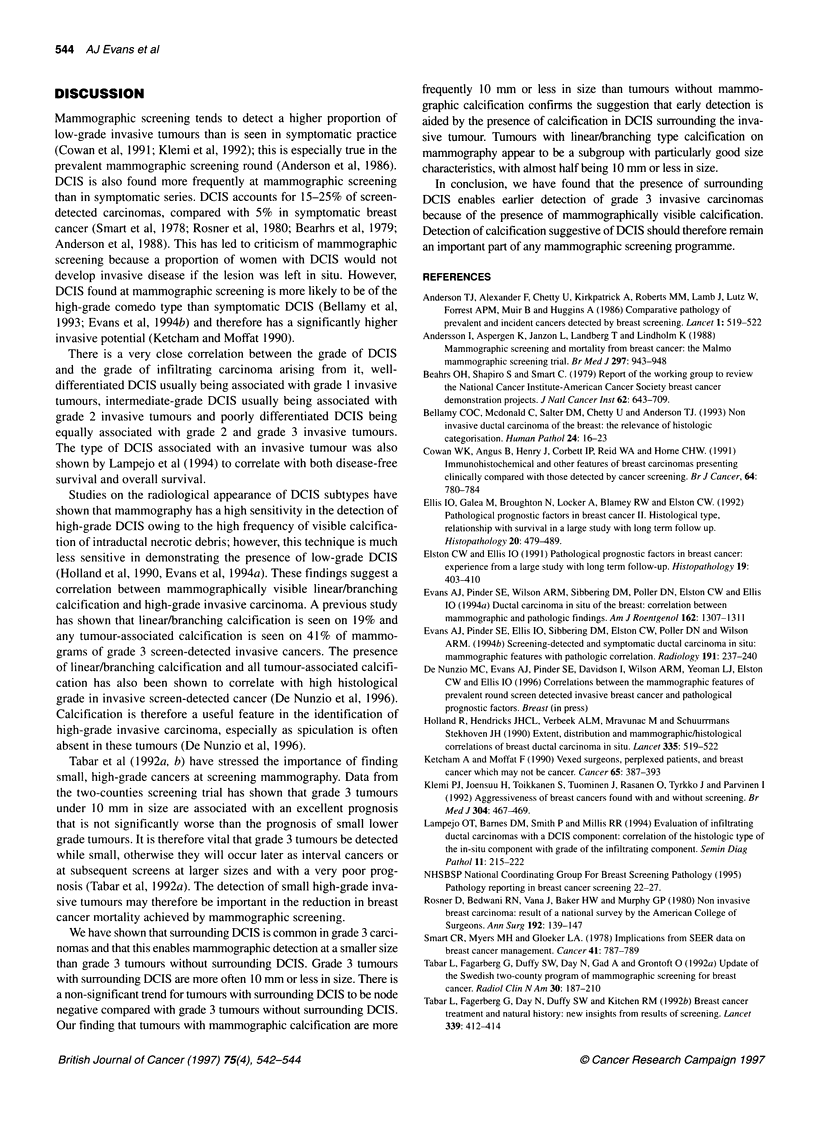# The detection of ductal carcinoma in situ at mammographic screening enables the diagnosis of small, grade 3 invasive tumours.

**DOI:** 10.1038/bjc.1997.94

**Published:** 1997

**Authors:** A. J. Evans, S. E. Pinder, D. R. Snead, A. R. Wilson, I. O. Ellis, C. W. Elston

**Affiliations:** Department of Radiology, Nottingham City Hospital, UK.

## Abstract

This study was carried out to assess the frequency of ductal carcinoma in situ (DCIS) occurring within and surrounding grade 3 invasive tumours and the effect of its detection on size and nodal stage of invasive carcinomas at mammographic detection. Grade 3 tumours with either no associated DCIS or DCIS only within the invasive component were significantly larger in size than tumours with surrounding DCIS (P < 0.02) and were less likely to be under or equal to 10 mm in size (0% or 13% vs 30% respectively, P < 0.02). Tumours with mammographic calcification were more likely to be less than or equal to 10 mm in size than non-calcific tumours (32% vs 11% respectively, P < 0.05). This was because of the high frequency of tumours less than or equal to 10 mm in size in the linear/branching calcification group. Tumours showing calcification without a mass also appear to be a group with good prognostic features, with a mean size of 13 mm, 33% being 10 mm or less in size and only 17% being node positive. We have found that the presence of surrounding DCIS enables earlier detection of grade 3 invasive carcinomas because of the presence of mammographically visible calcification. Detection of calcification suggestive of DCIS should remain an important part of mammographic screening.


British Journal of Cancer (1997) 75(4), 542–544
© 1997 Cancer Research Campaign

### The detection of ductal carcinoma in situ at mammographic screening enables the diagnosis of small, grade 3 invasive tumours

**AJ Evans, SE Pinder, DRJ Snead, ARM Wilson, IO Ellis and CW Elston**

Departments of Radiology and Pathology, Nottingham City Hospital, Nottingham, UK

**Summary** This study was carried out to assess the frequency of ductal carcinoma in situ (DCIS) occurring within and surrounding grade 3 invasive tumours and the effect of its detection on size and nodal stage of invasive carcinomas at mammographic detection. Grade 3 tumours with either no associated DCIS or DCIS only within the invasive component were significantly larger in size than tumours with surrounding DCIS ($P<0.02$) and were less likely to be under or equal to 10 mm in size (0% or 13% *vs* 30% respectively, $P<0.02$). Tumours with mammographic calcification were more likely to be less than or equal to 10 mm in size than non-calcific tumours (32% *vs* 11% respectively, $P<0.05$). This was because of the high frequency of tumours less than or equal to 10 mm in size in the linear/branching calcification group. Tumours showing calcification without a mass also appear to be a group with good prognostic features, with a mean size of 13 mm, 33% being 10 mm or less in size and only 17% being node positive. We have found that the presence of surrounding DCIS enables earlier detection of grade 3 invasive carcinomas because of the presence of mammographically visible calcification. Detection of calcification suggestive of DCIS should remain an important part of mammographic screening.

**Keywords:** mammographic screening; DCIS; grade 3 breast cancer

### INTRODUCTION

Breast cancer detected by mammographic screening has a higher proportion of low-grade invasive carcinomas and ductal carcinoma in situ (DCIS) than symptomatic breast cancer (Cowan et al, 1991; Klemi et al, 1992). This has led some to suspect a degree of overdiagnosis, at least in the prevalent screening round, when such excellent-prognosis tumours are most frequently found (Anderson et al, 1986).

Tabar et al (1992*a,b*) have stressed the importance of detecting small high-grade invasive tumours at mammographic screening. This is because the prognosis of grade 3 tumours, if detected while less than 10 mm in size, is excellent and may not be significantly worse than the prognosis of small lower-grade tumours. It is important that grade 3 tumours be detected while small, before they present as either interval cancers or at subsequent screens at large sizes and with a very poor prognosis (Tabar et al, 1992*a*). The detection of small high-grade invasive tumours may therefore be important in the reduction in breast cancer mortality achieved by mammographic screening.

There is a strong association between the grade of DCIS and the grade of invasive tumour arising from it (Lampejo et al, 1994). High-grade DCIS, which is usually mammographically visible (Holland et al, 1990; Evans et al, 1994*a*), as calcification is frequently associated with high-grade invasive tumours. A previous study has shown calcification to be a common feature of grade 3 screen-detected tumours (De Nunzio et al, 1996). We therefore postulate that high-grade DCIS is commonly associated with grade 3 invasive tumours and that the presence of surrounding DCIS manifesting as calcification may aid the early diagnosis of grade 3 invasive tumours.

This study was carried out to assess the frequency of DCIS occurring within and surrounding grade 3 invasive tumours and the effect of its detection on size and nodal stage of invasive carcinomas at mammographic detection. Grading of invasive cancers, in this study, was performed using the Nottingham method (Elston et al, 1991). This technique involves semiquantitive evaluation of three morphological features: the percentage of tubule formation, the degree of nuclear pleomorphism and an accurate mitotic count using a defined field area.

### MATERIALS AND METHODS

The histopathology and mammographic findings of all patients with screen-detected grade 3 invasive breast carcinomas between 1988 and 1994 were reviewed. Sixty-one patients were included in the study. According to radiological opinion, the lesions were classified as 38% malignant, 35% probably malignant, 23% indeterminate and 4% probably benign. Fifty (86%) of 58 fine-needle aspiration cytologies performed were malignant, and seven(88%) of the eight core-cut biopsies performed were malignant.

The presence of a grade 3 invasive tumour was confirmed using the Nottingham method (Elston et al, 1991), its histological type established (Ellis et al, 1992) and its maximum invasive size measured. The presence or absence of any associated DCIS was ascertained and its histological subtype recorded (NHSBSP, 1995). If DCIS was present, it was established whether the DCIS was found only within 1 mm of the invasive tumour (minimal DCIS) or whether there was DCIS surrounding the invasive tumour. This classification was performed by two pathologists independently, and there was agreement in 94% of cases.

*Received 15 January 1996*
*Revised 8 August 1996*
*Accepted 29 August 1996*

*Correspondence to:* AJ Evans, Helen Garrod Breast Screening Unit, City Hospital, Hucknall Road, Nottingham NG5 1PB, UK

**Table 1** Histological size and nodal status of grade 3 tumours by associated DCIS

| DCIS status | No. of grade 3 tumours (%) | Size range (mm) | Median size (mm) | Mean size (mm) | No. of grade 3 tumours <10 mm (%) | Nodal stage: Stage 1 | Stage 2 | Stage 3 | Stage 2 or 3 |
|---|---|---|---|---|---|---|---|---|---|
| No DCIS | 13 (21) | 13–35 | 20 | 20 | 0 | 7 | 3 | 2 | 5 (42)[a] |
| Minimal DCIS | 15 (25) | 10–30 | 21 | 20 | 2 (13) | 8 | 5 | 1 | 6 (43) |
| Surrounding DCIS | 33 (54) | 1.5–33 | 14 | 15 | 10 (30) | 23 | 7 | 2 | 9 (28) |

[a]Numbers in parenthese are percentages.

**Table 2** Histological size and nodal status of grade 3 tumours according to mammographic appearance

| Mammographic appearance | No. of grade 3 tumours (%) | Size range (mm) | Median size (mm) | Mean size (mm) | No. of grade 3 tumours ≤ 10 mm (%) | Nodal stage: Stage 1 | Stage 2 | Stage 3 | Stage 2 or 3 |
|---|---|---|---|---|---|---|---|---|---|
| Granular/punctate calcification | 11 (18) | 6–28 | 19 | 16 | 2 (18) | 6 | 5 | 0 | 5 (43)[a] |
| Linear/branching calcification | 14 (23) | 1.5–33 | 12 | 15 | 6 (43) | 10 | 3 | 1 | 4 (29) |
| All suspicious calcification | 25 (42) | 1.5–33 | 19 | 15 | 8 (32) | 16 | 8 | 1 | 9 (36) |
| Calcification no mass | 6 (10) | 1.5–28 | 13 | 13 | 2 (33) | 5 | 1 | 0 | 1 (17) |
| Calcification with mass | 19 (32) | 6–33 | 19 | 19 | 5 (26) | 11 | 7 | 1 | 8 (42) |
| Mass without calcification | 35 (58) | 5–35 | 18 | 18 | 4 (11) | 22 | 7 | 4 | 11 (33) |

[a]Numbers in parentheses are percentages.

Lymph node stage was assessed by histological examination of surgical axillary node sampling and internal mammary node sampling in medial tumours. Nodal stage was subdivided into three groups: stage 1, no node involvement; stage 2, three nodes or fewer with metastatic involvement; and stage 3 four or more nodes with metastatic involvement. Patients in whom lymph node sampling was not performed were excluded from analysis of lymph node stage.

The diagnostic mammograms were reviewed by a radiologist who knew that the patient had a grade 3 carcinoma but was unaware of any other pathological data. Abnormalities were classified as masses, linear/branching calcification or punctate/granular calcification. The size and nodal stage of the invasive tumours in the different groups were compared. The significance of differences between groups was established using the chi-square, Fisher's exact and Mann–Whitney *U*-tests.

### RESULTS

The pathology slides of 61 screen-detected grade 3 cancers were available for review. The mean histological invasive size was 16.9 mm. Eighty-two percent of the cancers were ductal carcinomas of no special type. Size and nodal status were unavailable in one case because the tumour was locally advanced and the patient received non-operative therapy. Nodal status was not available in two other cases.

Thirteen (21%) tumours had no associated DCIS, 15 (25%) had DCIS confined to within the invasive component (minimal DCIS) and 33 (54%) had DCIS surrounding the invasive tumour. Ninety-five percent of associated DCIS were of high nuclear grade, 2% were of intermediate nuclear grade and 2% of low nuclear grade. The invasive histological sizes and nodal characteristics are shown in Table 1. Grade 3 tumours with either no associated DCIS or DCIS only within the invasive component were significantly larger in size than tumours with surrounding DCIS ($P<0.02$) and were less likely to be under or equal to 10 mm in size (0% or 13% *vs* 30% respectively, $P<0.02$).

There was a trend for tumours with surrounding DCIS to have less frequent nodal involvement than the other two groups, but this did not reach statistical significance (28% *vs* 42% and 43% respectively). No mammographic calcification was seen in the no DCIS group; however it was seen in 40% of the minimal DCIS group and 58% of the surrounding DCIS group.

Fourteen (23%) tumours showed linear/branching calcification, 11(18%) tumours showed punctate/granular calcification and 35 (58%) tumours showed masses without calcification on mammography (54% ill-defined masses, 37% spiculate masses, 8% architectural distortions, 5% developing density and 5% asymmetric density). One tumour was detected clinically by the radiographer below the inframammary fold and was therefore not imaged by mammography. The histological size and lymph node stage characteristics, according to mammographic appearance, are shown in Table 2. Grade 3 tumours showing mammographic calcification were not significantly smaller overall than non-calcific tumours. Calcific tumours were, however, more likely to be less than or equal to 10 mm in size than non-calcific tumours (32% *vs* 11% respectively, $P<0.05$). This was because of the high frequency of tumours less than or equal to 10 mm in size in the linear/branching calcification group. This group represents only 23% of all the tumours but contained 50% of all such small tumours. 43% of tumours showing linear/branching calcification were less than or equal to 10 mm in size compared with 13% in those tumours not showing linear/branching calcification mammographically.

Tumours showing calcification without a mass also appear to be a group with particularly good prognostic features, with a mean size of 13 mm, 33% being 10 mm or less in size and only 17% being node positive (Table 2).

### DISCUSSION

Mammographic screening tends to detect a higher proportion of low-grade invasive tumours than is seen in symptomatic practice (Cowan et al, 1991; Klemi et al, 1992); this is especially true in the prevalent mammographic screening round (Anderson et al, 1986). DCIS is also found more frequently at mammographic screening than in symptomatic series. DCIS accounts for 15–25% of screen-detected carcinomas, compared with 5% in symptomatic breast cancer (Smart et al, 1978; Rosner et al, 1980; Bearhrs et al, 1979; Anderson et al, 1988). This has led to criticism of mammographic screening because a proportion of women with DCIS would not develop invasive disease if the lesion was left in situ. However, DCIS found at mammographic screening is more likely to be of the high-grade comedo type than symptomatic DCIS (Bellamy et al, 1993; Evans et al, 1994*b*) and therefore has a significantly higher invasive potential (Ketcham and Moffat 1990).

There is a very close correlation between the grade of DCIS and the grade of infiltrating carcinoma arising from it, well-differentiated DCIS usually being associated with grade 1 invasive tumours, intermediate-grade DCIS usually being associated with grade 2 invasive tumours and poorly differentiated DCIS being equally associated with grade 2 and grade 3 invasive tumours. The type of DCIS associated with an invasive tumour was also shown by Lampejo et al (1994) to correlate with both disease-free survival and overall survival.

Studies on the radiological appearance of DCIS subtypes have shown that mammography has a high sensitivity in the detection of high-grade DCIS owing to the high frequency of visible calcification of intraductal necrotic debris; however, this technique is much less sensitive in demonstrating the presence of low-grade DCIS (Holland et al, 1990, Evans et al, 1994*a*). These findings suggest a correlation between mammographically visible linear/branching calcification and high-grade invasive carcinoma. A previous study has shown that linear/branching calcification is seen on 19% and any tumour-associated calcification is seen on 41% of mammograms of grade 3 screen-detected invasive cancers. The presence of linear/branching calcification and all tumour-associated calcification has also been shown to correlate with high histological grade in invasive screen-detected cancer (De Nunzio et al, 1996). Calcification is therefore a useful feature in the identification of high-grade invasive carcinoma, especially as spiculation is often absent in these tumours (De Nunzio et al, 1996).

Tabar et al (1992*a, b*) have stressed the importance of finding small, high-grade cancers at screening mammography. Data from the two-counties screening trial has shown that grade 3 tumours under 10 mm in size are associated with an excellent prognosis that is not significantly worse than the prognosis of small lower grade tumours. It is therefore vital that grade 3 tumours be detected while small, otherwise they will occur later as interval cancers or at subsequent screens at larger sizes and with a very poor prognosis (Tabar et al, 1992*a*). The detection of small high-grade invasive tumours may therefore be important in the reduction in breast cancer mortality achieved by mammographic screening.

We have shown that surrounding DCIS is common in grade 3 carcinomas and that this enables mammographic detection at a smaller size than grade 3 tumours without surrounding DCIS. Grade 3 tumours with surrounding DCIS are more often 10 mm or less in size. There is a non-significant trend for tumours with surrounding DCIS to be node negative compared with grade 3 tumours without surrounding DCIS. Our finding that tumours with mammographic calcification are more frequently 10 mm or less in size than tumours without mammographic calcification confirms the suggestion that early detection is aided by the presence of calcification in DCIS surrounding the invasive tumour. Tumours with linear/branching type calcification on mammography appear to be a subgroup with particularly good size characteristics, with almost half being 10 mm or less in size.

In conclusion, we have found that the presence of surrounding DCIS enables earlier detection of grade 3 invasive carcinomas because of the presence of mammographically visible calcification. Detection of calcification suggestive of DCIS should therefore remain an important part of any mammographic screening programme.

### REFERENCES

Anderson TJ, Alexander F, Chetty U, Kirkpatrick A, Roberts MM, Lamb J, Lutz W, Forrest APM, Muir B and Huggins A (1986) Comparative pathology of prevalent and incident cancers detected by breast screening. *Lancet* **1**: 519–522

Andersson I, Aspergen K, Janzon L, Landberg T and Lindholm K (1988) Mammographic screening and mortality from breast cancer: the Malmo mammographic screening trial. *Br Med J* **297**: 943–948

Beahrs OH, Shapiro S and Smart C. (1979) Report of the working group to review the National Cancer Institute-American Cancer Society breast cancer demonstration projects. *J Natl Cancer Inst* **62**: 643–709.

Bellamy COC, Mcdonald C, Salter DM, Chetty U and Anderson TJ. (1993) Non invasive ductal carcinoma of the breast: the relevance of histologic categorisation. *Human Pathol* **24**: 16–23

Cowan WK, Angus B, Henry J, Corbett IP, Reid WA and Horne CHW. (1991) Immunohistochemical and other features of breast carcinomas presenting clinically compared with those detected by cancer screening. *Br J Cancer*, **64**: 780–784

Ellis IO, Galea M, Broughton N, Locker A, Blamey RW and Elston CW. (1992) Pathological prognostic factors in breast cancer II. Histological type, relationship with survival in a large study with long term follow up. *Histopathology* **20**: 479–489.

Elston CW and Ellis IO (1991) Pathological prognostic factors in breast cancer: experience from a large study with long term follow-up. *Histopathology* **19**: 403–410

Evans AJ, Pinder SE, Wilson ARM, Sibbering DM, Poller DN, Elston CW and Ellis IO (1994*a*) Ductal carcinoma in situ of the breast: correlation between mammographic and pathologic findings. *Am J Roentgenol* **162**: 1307–1311

Evans AJ, Pinder SE, Ellis IO, Sibbering DM, Elston CW, Poller DN and Wilson ARM. (1994*b*) Screening-detected and symptomatic ductal carcinoma in situ: mammographic features with pathologic correlation. *Radiology* **191**: 237–240

De Nunzio MC, Evans AJ, Pinder SE, Davidson I, Wilson ARM, Yeoman LJ, Elston CW and Ellis IO (1996) Correlations between the mammographic features of prevalent round screen detected invasive breast cancer and pathological prognostic factors. *Breast* (in press)

Holland R, Hendricks JHCL, Verbeek ALM, Mravunac M and Schuurrmans Stekhoven JH (1990) Extent, distribution and mammographic/histological correlations of breast ductal carcinoma in situ. *Lancet* **335**: 519–522

Ketcham A and Moffat F (1990) Vexed surgeons, perplexed patients, and breast cancer which may not be cancer. *Cancer* **65**: 387–393

Klemi PJ, Joensuu H, Toikkanen S, Tuominen J, Rasanen O, Tyrkko J and Parvinen I (1992) Aggressiveness of breast cancers found with and without screening. *Br Med J* **304**: 467–469.

Lampejo OT, Barnes DM, Smith P and Millis RR (1994) Evaluation of infiltrating ductal carcinomas with a DCIS component: correlation of the histologic type of the in-situ component with grade of the infiltrating component. *Semin Diag Pathol* **11**: 215–222

NHSBSP National Coordinating Group For Breast Screening Pathology (1995) Pathology reporting in breast cancer screening 22–27.

Rosner D, Bedwani RN, Vana J, Baker HW and Murphy GP (1980) Non invasive breast carcinoma: result of a national survey by the American College of Surgeons. *Ann Surg* **192**: 139–147

Smart CR, Myers MH and Gloeker LA. (1978) Implications from SEER data on breast cancer management. *Cancer* **41**: 787–789

Tabar L, Fagarberg G, Duffy SW, Day N, Gad A and Grontoft O (1992*a*) Update of the Swedish two-county program of mammographic screening for breast cancer. *Radiol Clin N Am* **30**: 187–210

Tabar L, Fagerberg G, Day N, Duffy SW and Kitchen RM (1992*b*) Breast cancer treatment and natural history: new insights from results of screening. *Lancet* **339**: 412–414

© Cancer Research Campaign 1997